# Induction of microglial toll-like receptor 4 by prothrombin kringle-2: a potential pathogenic mechanism in Parkinson’s disease

**DOI:** 10.1038/srep14764

**Published:** 2015-10-06

**Authors:** Won-Ho Shin, Min-Tae Jeon, Eunju Leem, So-Yoon Won, Kyoung Hoon Jeong, Sang-Joon Park, Catriona McLean, Sung Joong Lee, Byung Kwan Jin, Un Ju Jung, Sang Ryoung Kim

**Affiliations:** 1 School of Life Sciences, Kyungpook National University, Daegu 702-701, Korea; 2 BK21 plus KNU Creative BioResearch Group, Kyungpook National University, Daegu 702-701, Korea; 3 College of Veterinary Medicine, Kyungpook National University, Daegu 702-701, Korea; 4 Next-generation pharmaceutical research center, Korea Institute of Toxicology, Korea Research Institute of Chemical Technology, Daejeon, 305-343, Korea; 5 Department of Biochemistry and Medical Research Center, Chungbuk National University College of Medicine, Cheongju 361-763, Korea; 6 Victorian Brain Bank Network, Florey Institute of Neuroscience and Mental Health, The University of Melbourne, Melbourne, VIC 3004, Australia; 7 Department of Anatomical Pathology, Alfred Hospital, Melbourne, VIC 3004, Australia; 8 Department of Neuroscience and Physiology, School of Dentistry, Seoul National University, Seoul, 110-749, Korea; 9 Dental Research Institute, School of Dentistry, Seoul National University, Seoul, 110-749, Korea; 10 Department of Biochemistry & Molecular Biology, School of Medicine, Kyung Hee University, Seoul 130-701, Korea; 11 Neurodegeneration Control Research Center, School of Medicine, Kyung Hee University, Seoul 130-701, Korea; 12 Department of Food Science and Nutrition, Pukyong National University, Busan 608-737, Korea; 13 Institute of Life Science & Biotechnology, Kyungpook National University, Daegu 702-701, Korea; 14 Brain Science and Engineering Institute, Kyungpook National University, Daegu 700-842, Korea

## Abstract

Microglia-mediated neuroinflammation may play an important role in the initiation and progression of dopaminergic (DA) neurodegeneration in Parkinson’s disease (PD), and toll-like receptor 4 (TLR4) is essential for the activation of microglia in the adult brain. However, it is still unclear whether patients with PD exhibit an increase in TLR4 expression in the brain, and whether there is a correlation between the levels of prothrombin kringle-2 (pKr-2) and microglial TLR4. In the present study, we first observed that the levels of pKr-2 and microglial TLR4 were increased in the substantia nigra (SN) of patients with PD. In rat and mouse brains, intranigral injection of pKr-2, which is not directly toxic to neurons, led to the disruption of nigrostriatal DA projections. Moreover, microglial TLR4 was upregulated in the rat SN and in cultures of the BV-2 microglial cell line after pKr-2 treatment. In TLR4-deficient mice, pKr-2-induced microglial activation was suppressed compared with wild-type mice, resulting in attenuated neurotoxicity. Therefore, our results suggest that pKr-2 may be a pathogenic factor in PD, and that the inhibition of pKr-2-induced microglial TLR4 may be protective against degeneration of the nigrostriatal DA system *in vivo*.

The defining neuropathological feature of Parkinson’s disease (PD) is the loss of nigrostriatal dopaminergic (DA) projections, which results in a biochemical decrease in striatal dopamine levels^1^,^2^. Although our understanding of the cause of PD remains elusive, accumulating evidence suggests that neuroinflammatory processes are crucial for the initiation and progression of PD, and that microglia, the resident immune cells of the brain, are important mediators of brain inflammation, which has been implicated in neurotoxicity^3^,^4^.

Prothrombin kringle-2 (pKr-2), is a domain of prothrombin that is generated by active thrombin, which itself originates from prothrombin cleavage^5^,^6^. Recently, we reported that pKr-2 was able to induce the death of DA neurons in the rat substantia nigra (SN) through microglial activation, even though pKr-2 itself was not directly toxic to neurons^7^. These results suggest that limiting pKr-2-induced microglial activation may be an effective therapeutic strategy for protecting DA neurons in the adult brain. However, it is unknown whether pKr-2 levels are upregulated in the brains of patients with PD. Moreover, the potential degenerative effects of increased pKr-2 expression on nigrostriatal DA projections have not yet been established.

Toll-like receptors (TLRs) are pattern-recognition receptors that recognize specific pathogen-associated molecular signatures and subsequently initiate inflammatory and immune responses^8^,^9^. Toll-like receptor 4 (TLR4) recognizes various ligands such as lipopolysaccharide, envelope proteins, heat-shock proteins, fibrinogen, and hyaluronan^9^,^10^, and its activation in immune cells induces an increase in inflammatory cytokines^11^,^12^. In the brain, microglia are important cells expressing TLR4, and TLR4-mediated signaling pathways within microglia stimulate the production of neurotoxic inflammatory cytokines^13^,^14^,^15^. Increased levels of such cytokines have been implicated in the pathology of many diseases of the central nervous system, such as cerebral ischemia^16^, traumatic brain injury^17^, Alzheimer’s disease (AD)^18^, and PD^19^. However, it remains unclear whether the levels of TLR4 are indeed increased in the brains of patients with PD, and whether TLR4 induction in microglia is necessary for pKr-2-mediated neurotoxicity. In the present study, we therefore investigated whether patients with PD exhibit increases in pKr-2 and TLR4 expression in the SN compared with age-matched controls. We then determined whether there is a correlation between pKr-2-induced neurotoxicity and microglial TLR4 expression in rodent models, and assessed the specific effects of intranigral injection of pKr-2, which caused disruption of nigrostriatal DA projections in the adult brain.

## Results

### Activation of microglia and an increase in microglial TLR4 in the SN of patients with PD

**Figure 1:**
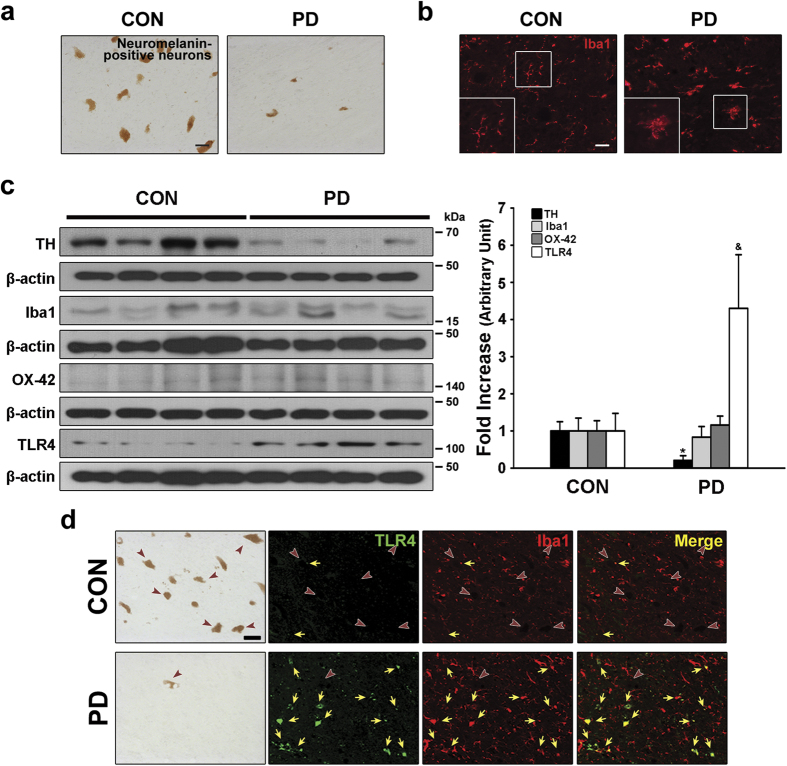
Morphological changes in microglia and an increase in microglial TLR4 in the SN of PD brains. (**a**) Patients with PD showed a decrease in neuromelanin-positive neurons (DA neurons) in the SN compared with age-matched controls. The pictures show a representative SN section from each group. Scale bar, 30 μm. (**b**) In SN sections from patients with PD, many microglia stained with anti-Iba1 showed an activated morphology, characterized by enlarged cell bodies with short processes. Insets show representative resting and activated microglia in the SN of an age-matched control and a patient with PD, respectively. Scale bar, 20 μm. (**c**) Western blot analysis showed that TH expression was significantly decreased in the SN samples from patients with PD compared with age-matched controls (CON), but there was not a significant difference in the levels of either Iba1 or OX-42. ^*^p = 0.001 *vs.* CON (*n* = 4, each group). In addition, western blot analysis of TLR4 showed that patients with PD exhibited a significant increase in its expression in the SN compared with CON. ^&^*p* = 0.005 *vs.* CON (*n* = 4, each group). (**d**) Immunofluorescence double staining for Iba1 (microglia, red) and TLR4 (green) in the SN of patients with PD and age-matched controls. Brown arrowheads and yellow arrows indicate the location of DA neurons and TLR4-positive cells merged with microglia, respectively, in SN sections of human brain. Increases in TLR4 expression and activated microglia were observed in the SN of patients with PD compared with age-matched controls, and increased TLR4 expression was mainly localized within microglia. Scale bar, 30 μm.

Microglial activation in the brains of patients with PD has been well described in previous reports^3^,^4^. To assess microglial activation in the SN of the patients with PD used in the current study (Table 1), we examined the morphological changes in microglia by immunostaining with anti-ionized calcium-binding adapter molecule 1 (Iba1). Our observations showed that decreases in neuromelanin-positive neurons (DA neurons) were confirmed in SN sections from patients with PD compared with sections from age-matched controls (Fig. 1a), and many microglia that were stained with anti-Iba1 had an activated morphology characterized by enlarged cell bodies with short processes (Fig. 1b). Consistent with decreases in neuromelanin-positive neurons, western blot analysis showed that tyrosine hydroxylase (TH) expression, a marker for DA neurons, was significantly decreased in the SN of patients with PD compared with age-matched controls (*p* = 0.001, Fig. 1c). However, the total level of Iba1 and OX-42, which also serves as a marker of microglial levels, was not significantly different between patients with PD and age-matched controls (Fig. 1c), even though there was a significant increase in tumor necrosis factor-alpha (TNF-α) as a neurotoxic inflammatory cytokine, which could be produced by activated microglia, in the SN of patients with PD compared with age-matched controls (Extended data Fig. 1). These observations suggest that microglia-mediated neuroinflammation, which is capable of inducing neurodegeneration, may be caused by the activation state of the microglia, but not by the total number of microglia in the SN of human brains.

TLR4 is a crucial receptor for microglia-mediated neuroinflammation^13^,^14^,^15^, and its activation contributes to the production of neurotoxic inflammatory cytokines, which results in neurodegeneration in the adult brain^16^,^17^,^18^,^19^. However, it is still unclear whether the levels of microglial TLR4 are increased in the brains of patients with PD. To assess the levels of TLR4 expression in PD brains and controls, we measured its expression by western blotting and performed immunofluorescence double staining for Iba1 and TLR4. Western blotting indicated that there was a significant increase in TLR4 expression in SN samples from individuals with PD compared with age-matched controls (*p* = 0.005, Fig. 1c), even though there was no significant increase in the overall levels of Iba1 and OX-42 in the SN of patients with PD (Fig. 1c). Moreover, immunofluorescence staining confirmed the increase in TLR4 expression observed by western blotting, and further demonstrated that TLR4 was mainly localized within Iba1-positive cells (marked with yellow arrows, Fig. 1d), suggesting that there was a significant increase in microglial TLR4 in the SN of patients with PD compared with age-matched controls.

### Increase in pKr-2 expression in the SN of patients with PD

It is largely unknown whether there are any endogenous molecules capable of inducing neurodegeneration through microglial activation without a direct neurotoxic effect. We recently reported that pKr-2 was able to induce the death of DA neurons in the rat SN through microglial activation, even though pKr-2 itself was not directly toxic to neurons^7^. However, it is still unclear whether pKr-2 levels are upregulated in the brains of patients with PD, and whether an increase in pKr-2 expression induces degeneration of the nigrostriatal DA system in the adult brain.

**Figure 2:**
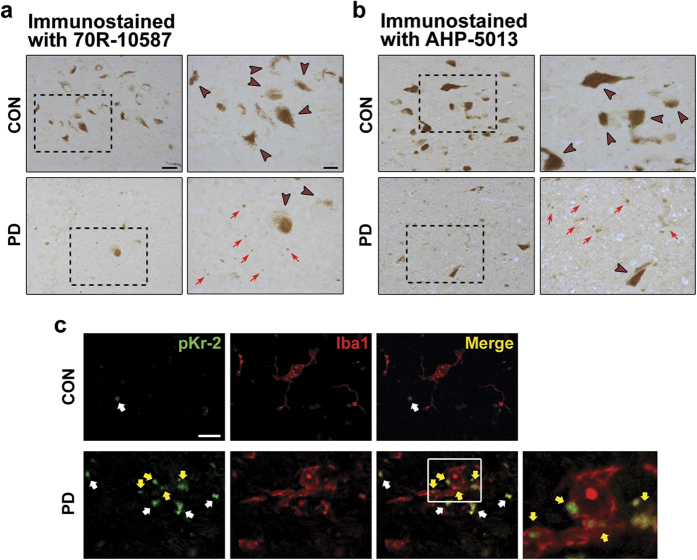
Increase in pKr-2 expression in the SN of PD brains. (**a**,**b**) SN tissues from patients with PD and age-matched controls were immunostained with anti-pKr-2 ((**a**) 70R-10587) or anti-prothrombin, which recognizes an epitope in the kringle-2 domain of prothrombin ((**b**) AHP-5013). Brown arrowheads and red arrows in the higher magnification images within each dotted rectangle indicate neuromelanin-positive neurons (DA neurons) and pKr-2 immunoreactivity, respectively, in the SN of human brains. Note that sections from patients with PD showed an increase in pKr-2 expression in the SN compared with age-matched controls. Scale bars, 40 μm and 20 μm, respectively. (**c**) Immunofluorescence staining showed that some of increased pKr-2 (green), which is marked with yellow arrows in the SN from PD-affected brains, was localized within Iba1-positive cells (red). All arrows indicate pKr-2 immunoreactivity. Scale bar, 10 μm.

To first evaluate whether there is an increase in pKr-2 expression in the SN of patients with PD, we compared immunohistochemical staining of pKr-2 in human postmortem SN tissues from control brains and PD brains. Specifically, the sections were immunostained with either an antibody raised against prothrombin fragment 2, which is a synonym for pKr-2 (70R-10587), or an antibody raised against prothrombin, which recognizes an epitope in the kringle-2 domain of prothrombin (AHP-5013). The results for both antibodies demonstrated increased immunoreactivity in SN sections from patients with PD compared with age-matched control sections (Red arrows, Fig. 2a,b). In addition to immunohistochemical staining, western blotting using the AHP-5013 antibody indicated that pKr-1–2 levels were significantly increased in the SN of PD brains compared with those of age matched controls (*p* = 0.018, Extended data Fig. 2), suggesting that pKr-2 levels might be increased in the SN of patients with PD. Moreover, immunofluorescence staining confirmed the increase in pKr-2 expression in the SN of patients with PD (marked with arrows, Fig. 2c), and further demonstrated that some of the pKr-2 expression was localized within Iba1-positive cells (marked with yellow arrows, Fig. 2c). This suggests that pKr-2 might be translocated within microglia, even though further research is needed to elucidate the mechanisms involved in the intracellular translocation of pKr-2 in the adult brain.

### Upregulation of pKr-2 disrupts nigrostriatal DA projections in the adult brain

**Figure 3:**
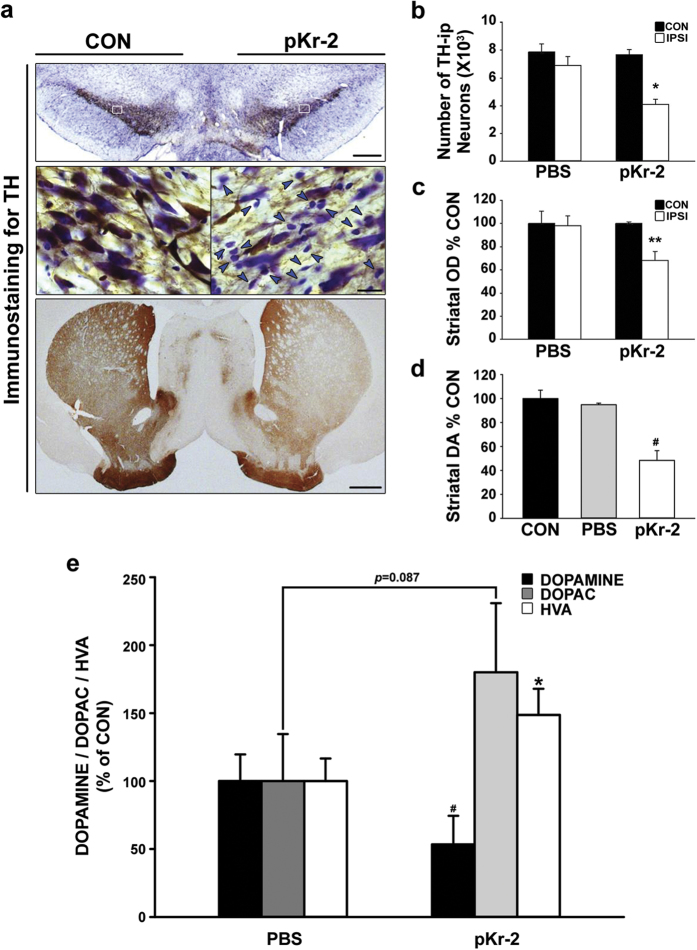
Increase in pKr-2 expression disrupts the nigrostriatal DA system in adult rat brains. (**a**) One week post-injection of pKr-2 into the SN, degeneration of nigrostriatal DA projections in the rat brain was apparent in coronal sections from the SN and STR immunostained with anti-TH. Many more small-sized, non-neuronal cells stained with cresyl violet (marked with blue arrowheads in the high-magnification image of the SN), were observed in the SN of animals treated with pKr-2 compared with intact controls. Scale bars, 500 μm and 20 μm for the SN, and 1000 μm for the STR. (**b**) A histogram showing results for the quantification of DA neurons in the SN. *p = 0.002 *vs.* contralateral control side (*n* = 5, each group). (**c**) The OD of TH-ip fibers in the STR. **p = 0.026 *vs.* contralateral control side (*n* = 5, each group). (**d**) The amount of dopamine in rat striatal tissues was measured by ELISA, and the levels were quantitatively expressed as a percentage of the value for the contralateral control for each sample. ^#^*p* < 0.001 *vs.* contralateral/PBS controls (*n* = 4, each group). (**e**) HPLC analysis of dopamine and its metabolites, DOPAC and HVA in the STR of rat brains shows that pKr-2 treatment significantly reduced striatal dopamine levels compared with PBS controls (*p* = 0.018; *n* = 4, each group). By contrast, the levels of striatal HVA were significantly increased by pKr-2 treatment compared with PBS controls (*p* = 0.029), and the levels of striatal DOPAC also showed a similar pattern in pKr-2-treated rats, even though this was not significantly different compared with PBS controls (*p* = 0.087).

To investigate whether upregulation of pKr-2 contributes to the degeneration of nigrostriatal DA projections, we unilaterally injected pKr-2 (48 μg in 4 μL) or vehicle (phosphate-buffered saline [PBS], 4 μL) into the SN of rats, as previously described^7^. Similar to our previous study^7^, a significant loss of TH-immunopositive (ip) neurons and fibers was observed in the SN and striatum (STR), respectively, a week after injection of pKr-2 (Fig. 3a). In addition to the observed neurotoxicity, there were more non-neuronal cells stained with cresyl violet in the pKr-2-treated SN than in the intact control SN (blue arrowheads in the high-magnification image of the SN, Fig. 3a), suggesting microglial activation by pKr-2 treatment^7^. Quantitatively, vehicle (PBS)-treated controls showed no signs of neurotoxicity (Fig. 3b,c). However, the number of TH-ip neurons and the optical density (OD) of striatal TH were significantly reduced by 47% (*p* = 0.002, Fig. 3b) and 32% (*p* = 0.026, Fig. 3c), respectively, in pKr-2-treated brain tissue compared with contralateral controls. Similar to the immunostaining data for TH-ip fibers in the STR, the levels of striatal dopamine measured using an enzyme-linked immunosorbent assay (ELISA) seven days after injection of pKr-2 were significantly decreased compared with controls (*p* < 0.001, Fig. 3d). To clarify the changes in striatal dopamine and its metabolites, 3,4-dihydroxyphenylacetic acid (DOPAC) and homovanillic acid (HVA), that were induced by pKr-2 treatment, we further examined the levels in the STR seven days after injection of pKr-2 using reversed-phase high performance liquid chromatography (HPLC) with an electrochemical detector^20^. HPLC analysis revealed that pKr-2 treatment reduced striatal dopamine levels to 47% of those in PBS-treated controls (*p* = 0.018, Fig. 3e). By contrast, the levels of striatal HVA were significantly increased by pKr-2 treatment compared with PBS controls (*p* = 0.029, Fig. 3e), and a similar pattern of increase in DOPAC was observed in pKr-2-treated rats compared with PBS-treated controls, even though this was not significantly different (*p* = 0.087, Fig. 3e). This suggested that there might be a compensatory increase in the turnover of dopamine following a partial nigrostriatal lesion^21^,^22^. Moreover, similar to the levels in PBS-treated controls, the increased levels of DA metabolites were reduced at 2 weeks after pKr-2 treatment (Extended data Fig. 3), indicating that there was functional loss in the nigrostriatal DA system. Taken together, these results suggest that an increase in pKr-2 expression may contribute to disruption of the nigrostriatal DA projections and act as a PD-inducing stimulus in the adult brain.

### pKr-2 treatment induces an increase in microglial TLR4 *in vitro* and *in vivo*

**Figure 4:**
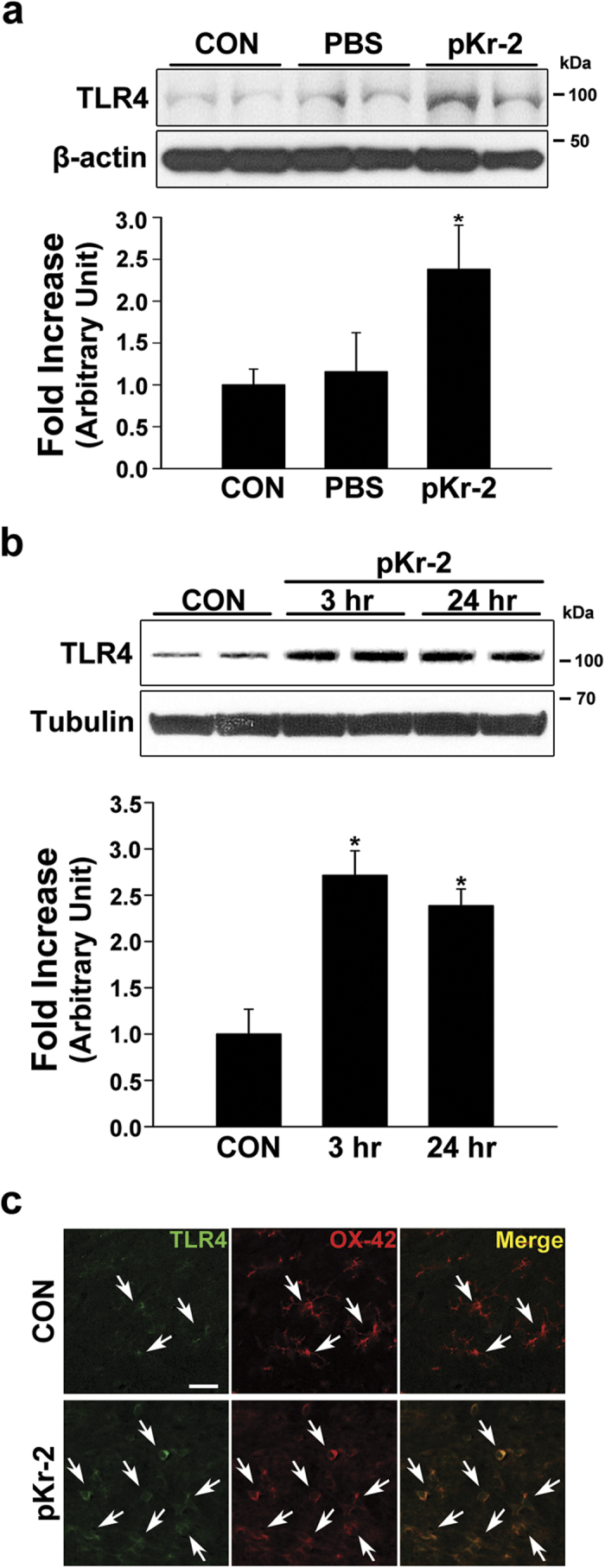
Upregulation of TLR4 in the SN of PD brains and pKr-2-treated rat brains. (**a**) Western blot analysis of TLR4 in BV-2 microglia cultures 6 h after PBS or pKr-2 treatment. The results showed that treatment with 50 μg/mL pKr-2^7^ significantly upregulated the levels of TLR4 compared with untreated controls and PBS-treated controls. *p = 0.002 and 0.006 *vs.* untreated controls and PBS-treated controls, respectively (*n* = 4, each group) (**b**) Western blot analysis of TLR4 in the SN of rat brains showed that its expression is increased 3 h and 24 h after intranigral injection of pKr-2 (48 μg in 4 μL) compared with PBS-treated controls (CON). The PBS-injected SN was analyzed 3 h after injection. *p < 0.001 *vs.* controls (*n* = 4, each group). (**c**) Immunofluorescence double staining for TLR4 and OX-42 (microglia) showed an increase in TLR4 expression colocalized within microglia (white arrows) in the pKr-2-treated SN compared with PBS controls. Scale bar, 20 μm.

Our results showed that the levels of pKr-2 (Fig. 2) and microglial TLR4 (Fig. 1c,d) were increased in the SN of patients with PD. However, it is still unclear whether there is a correlation between microglial TLR4 and pKr-2-induced neurotoxicity. To investigate this question, the levels of TLR4 were measured in cultures of the BV-2 microglial cell line by western blotting and in the SN of rat brains by western blotting and immunofluorescence double staining after pKr-2 exposure. In BV-2 microglial cell line cultures, treatment with 50 μg/mL pKr-2^7^ significantly upregulated the levels of TLR4 compared with untreated and PBS-treated controls (*p* = 0.002 and 0.006 *vs.* untreated and PBS-treated controls, respectively, Fig. 4a), suggesting that pKr-2 treatment directly increased microglial TLR4 expression. Consistent with the increase in TLR4 *in vitro*, an intranigral injection of pKr-2 (48 μg in 4 μL) significantly upregulated its expression in the rat SN 3 h and 24 h post-injection, compared with PBS-treated controls, as demonstrated by western blotting (*p* < 0.001, Fig. 4b) and immunofluorescence staining for TLR4 and OX-42 (Fig. 4c; 24 h post-injection). These results suggest that an increase in pKr-2 expression may lead to the induction of TLR4 in microglia in the SN of patients with PD.

### pKr-2-induced TLR4 contributes to degeneration of the nigrostriatal DA system in the adult brain

**Figure 5:**
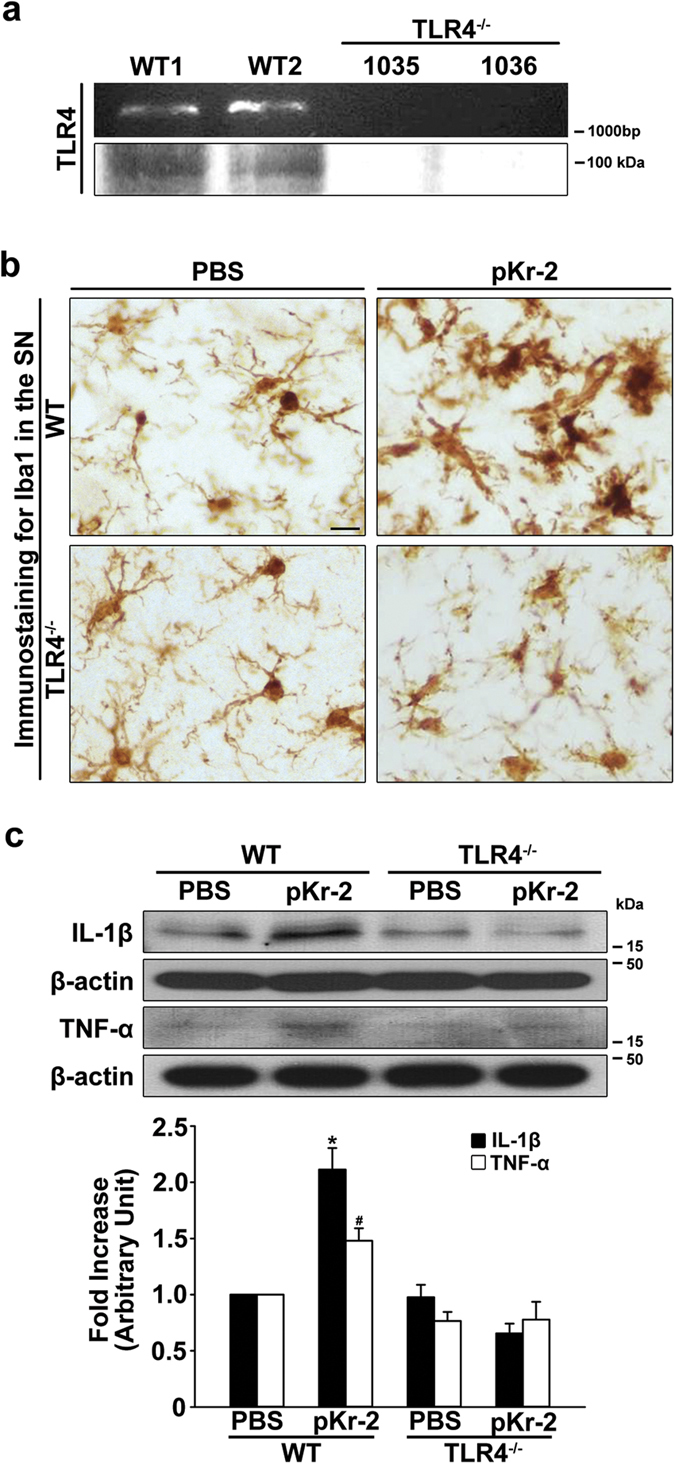
Inhibition of pKr-2-induced microglial activation in the SN of TLR4-deficient mice. (**a**) The loss of TLR4 in KO mice was confirmed by genotyping and western blot analysis of TLR4 expression. (**b**) Three days after intranigral injection of pKr-2 (24 μg in 2 μL) or PBS as a control, SN sections from WT and TLR4 KO mice were immunostained with anti-Iba1. The pKr-2-induced morphological changes to microglia, which indicate activated forms in the SN of WT mice, were reduced in TLR4 KO mice. (**c**) Consistent with increased microglial activation, western blot analysis showed significant increases in both IL-1β and TNF-α in the SN of pKr-2-treated WT mice compared with PBS-treated control mice. *p < 0.001 and ^#^*p* = 0.031, respectively, *vs.* PBS (*n* = 4, each group). However, there was no increase in IL-1β and TNF-α expression in the SN of pKr-2-treated TLR4 KO mice compared with PBS-treated controls.

To further ascertain whether pKr-2-induced microglial activation and neurotoxicity is correlated with the induction of TLR4, pKr-2 (24 μg in 2 μL) was injected into the SN of C57BL/6 wild-type (WT) control mice or TLR4 knockout (KO) mice, confirmed by genotyping and western blotting for TLR4 (Fig. 5a). PBS-treated WT mice, which showed no evidence of neurotoxicity and neuroinflammation, were used as controls for the effects of surgical damage to DA neurons and fibers in the mouse brain (Extended data Fig. 4a–c). To evaluate a possible link between pKr-2-induced TLR4 and microglial activation, SN sections from WT control and TLR4 KO mice were immunostained with Iba1 antibodies three days after an intranigral injection of PBS or pKr-2 (Fig. 5b) because pKr-2-induced microglial activation occurred prior to the apparent lesion of DA neurons in the SN *in vivo*^7^. In the PBS-treated WT SN, most Iba1-ip microglia displayed a resting morphology characterized by small cell bodies with long, thin and many ramified processes. However, pKr-2 treatment in WT mice caused microglial activation, characterized by enlarged cell bodies with short processes. By contrast, the morphological transformation of microglia resulting from pKr-2 treatment was attenuated in TLR4-deficient mice. Consistent with the morphological activation of microglia, western blot analysis showed significant increases in neurotoxic inflammatory cytokines mediated by microglia, such as interleukin-1 beta (IL-1β) and TNF-α^7^, in the SN of WT mice three days after injection of pKr-2 compared with PBS-treated controls (*p* < 0.001 and *p* = 0.031, respectively, Fig. 5c); these effects were significantly diminished in TLR4-deficient mice. In addition, western blot analysis of Iba1 expression in the SN revealed a significant increase in its levels in the SN of WT mice at three days after injection of pKr-2 (*p* < 0.001 *vs*. PBS-treated controls, Extended data Fig. 5), and the increase was significantly diminished in TLR4-deficient mice compared with WT mice (*p* = 0.009 *vs*. pKr-2-treated WT mice, Extended data Fig. 5). PBS-treated WT control mice showed no increase in the levels of IL-1β and TNF-α (Extended data Fig. 4d).

**Figure 6:**
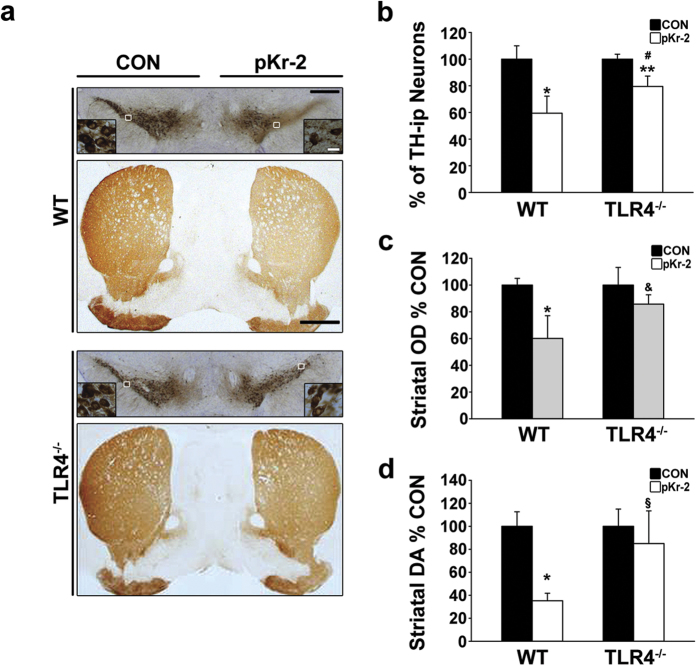
TLR4 deficiency attenuates pKr-2-induced neurotoxicity. (**a**) Mice received an intranigral injection of pKr-2 (24 μg in 2 μL), and TH immunostaining was assessed one week after treatment. The pKr-2 treatment induced a decrease in TH-ip neurons and fibers in the SN and STR, respectively, in WT mice. However, neurotoxicity was apparently attenuated in TLR4 KO mice compared with WT mice. Scale bars, 500 μm and 20 μm for the SN, and 1000 μm for the STR. (**b**) The preservation of DA neurons in the SN. The number of DA neurons in the SN was quantitatively expressed as a percentage of the value for the contralateral control side in each group. *p < 0.001 and **p = 0.037 *vs.* contralateral control side; ^#^*p* = 0.042 *vs.* pKr-2-treated WT (*n* = 4, each group). (**c**) The OD of TH-ip fibers in the STR. *p < 0.001 *vs.* contralateral control side; ^&^*p* = 0.014 *vs.* pKr-2-treated WT (*n* = 4, each group). (**d**) ELISA analysis of dopamine levels in the mouse STR. The levels were quantitatively expressed as a percentage of the value for the contralateral controls. *p < 0.001 *vs.* contralateral control side; ^§^*p* < 0.001 *vs.* pKr-2-treated WT (*n* = 4, each group).

Consistent with inhibition of pKr-2-induced microglial activation in TLR4-deficient mice (Fig. 5b,c), we observed that the subsequent loss of TH-ip neurons and fibers in the SN and STR, respectively, was significantly attenuated in TLR4 KO mice compared with WT mice (Fig. 6a). When quantified and expressed as a percentage of DA neurons in the counting area of the ipsilateral SN relative to the contralateral control SN one week after an intranigral injection of pKr-2, only 59% of DA neurons were preserved in the SN of the pKr-2-treated WT mice (*p* < 0.001 *vs.* intact controls, Fig. 6b). In contrast, 79% of DA neurons were preserved in TLR4 KO mice (*p* = 0.037 *vs.* intact controls, Fig. 6b) with a significant difference compared with the pKr-2-treated WT mice (*p* = 0.042, Fig. 6b). Moreover, there was no significant difference in the OD of TH-ip fibers between the contralateral control STR and pKr-2-treated ipsilateral STR of TLR4 KO mice (*p* = 0.249 *vs.* intact controls, Fig. 6c), even though this treatment induced a decrease in the OD by 40% in WT mice (*p* < 0.001 *vs.* intact controls, Fig. 6c). Consistent with the results for immunostaining with TH antibodies, the levels of striatal dopamine assessed by ELISA were significantly decreased in the pKr-2-treated WT mice (*p* < 0.001 *vs.* intact controls, Fig. 6d), but not in the TLR4 KO mice (*p* = 0.402 *vs.* intact controls, Fig. 6d). Taken together, our results suggest that pKr-2-induced microglial TLR4 may be a major mediator of pKr-2-induced neuroinflammation and neurotoxicity in the adult brain, even though TLR4 deficiency did not completely prevent the neurotoxicity induced by pKr-2 treatment.

## Discussion

Although the etiology of PD is not yet understood, accumulating evidence implicates microglia, which are the resident immune cells in the brain. They are crucial mediators of the brain inflammatory processes that lead to neurotoxicity, and microglial activation contributes to the initiation and progression of PD^4^,^7^,^23^. In the healthy brain, microglia are in a resting state that is characterized morphologically by small cell bodies with thin, ramified processes. In addition, resting microglia phenotypically show low expression of inflammatory molecules associated with immune functions^7^,^23^,^24^. However, under neuropathological conditions, activated microglia produce a spectrum of potentially neurotoxic molecules that contribute to the death of DA neurons^7^,^23^,^24^,^25^. In addition to neurotoxic cytokines, activated microglia generate reactive oxygen species, leading to oxidative stress in DA neurons^25^,^26^. Similar to these results, our observations showed that many microglia in the SN of patients with PD exhibited an increase in TLR4 expression with activated morphology (Fig. 1b–d). However, there was no significant difference in the total levels of Iba1 and OX-42, suggesting that the number of microglia might be similar between patients with PD and age-matched controls, as demonstrated by western blot analysis (Fig. 1c). These results suggest that the activation state of microglia rather than the number of microglia in the SN may mediate microglia-induced neurotoxicity towards DA neurons, and that control of microglial activation may be useful for preventing the degeneration of nigrostriatal DA projections in the adult brain.

Among the prothrombin domains, pKr-2 is produced by the cleavage of pKr-1-2 by active thrombin, which is generated following the activation of factor Xa in the prothrombinase complex^7^. Prothrombin, an endogenous source of thrombin, is expressed in the human brain^27^ and accumulates in the SN of human PD brains^28^. Additionally, increased thrombin levels have also been found in the brain in other neurodegenerative diseases such as AD^29^,^30^. Moreover, disruption of the blood-brain barrier (BBB), resulting in cerebrovascular disturbances, may be one of features shown in patients with PD and AD^31^; therefore, prothrombin and thrombin upregualtion may be mainly mediated by BBB breakdown, due to disruption of the tight junctions, in the brain of patients with PD. In a previous study, we showed that pKr-2 caused no direct neurotoxicity, but could activate microglia, and the resulting production of inflammatory cytokines from pKr-2-activated microglia contributed to the death of DA neurons *in vivo* and *in vitro*^7^. However, it was not determined whether pKr-2 expression is upregulated in the brains of patients with PD, and we did not assess the potential disruption of the nigrostriatal DA pathway, which is a representative phenotype of PD. Here, we present the first report showing that patients with PD have increases in pKr-2 and pKr-1-2 expression in the SN (Fig. 2a–c and Extended data Fig. 2, respectively), and the increase in pKr-2 expression disrupts the nigrostriatal DA system in the adult murine brain (Fig. 3,6 and Extended data Fig. 3). Moreover, some of pKr-2 expression were co-localized within activated microglia as shown in Fig. 2c, even though its expression was not localized within DA neurons (data not shown), suggesting that pKr-2 might be upregulated in neurodegenerative diseases due to an increase in active thrombin originated from blood, even though there have been no studies so far that have reported the upregulation of pKr-2 and cell types of pKr-2 production or secretion in human brains.

The pattern of TLR4 expression in the brain is controversial. Lehnardt *et al.* reported that TLR4 is expressed only in microglia^13^. By contrast, TLR4 expression in other brain cells such as astrocytes, oligodendrocytes, and neurons has been reported by many research groups^32^,^33^, and TLR4 may have different roles in glia and neurons^34^. However, there are many reports suggesting that microglia are important for TLR4-mediated immune responses, which may be involved in brain diseases such as AD^18^ and PD^19^. Moreover, an increase in TLR4 expression has been found in α-synuclein-overexpressing transgenic mice and patients with human multiple system atrophy^35^, suggesting the possibility that TLR4 is upregulated in the brains of patients with PD. This is consistent with results showing that upregulation of TLR4 contributes to neurotoxicity in 1-methyl-4-phenyl-1, 2, 3, 6-tetrahydropyridine (MPTP)-treated animal models of PD^36^,^37^,^38^. However, alterations in TLR4 expression in patients with PD had not yet been demonstrated, and it was unclear whether there are any endogenous molecules capable of inducing DA neurodegeneration through microglial activation via TLR4 induction, but in the absence of any direct neurotoxic effect.

To clarify whether TLR4 expression is increased in the brains of patients with PD, we measured its expression in the postmortem human SN from control and PD-affected brains by western blotting and immunofluorescence double staining. We found that that TLR4 levels were greater in the SN of patients with PD compared with age-matched controls (Fig. 1c,d), indicating that there is indeed an increase in TLR4 expression in PD brains, even though the total levels of microglia were not significantly different between patients with PD and age-matched controls (Fig. 1c). Moreover, the increased TLR4 expression was mainly localized within Iba1-positive microglia (Fig. 1d), suggesting that an increase in microglial TLR4 might be crucial for the pathogenesis of PD, and the discovery of endogenous molecules involved in the induction of microglial TLR4 might be useful for guiding the development of knowledge-based targeted therapeutics for PD. In the present study, we also found that pKr-2-induced neurotoxicity and microglial activation was correlated with the induction of TLR4 in microglia. Consistent with increases in both pKr-2 (Fig. 2a–c) and microglial TLR4 (Fig. 1c,d) in the SN of patients with PD, pKr-2 treatment increased microglial TLR4 in cultures of the BV-2 microglial cell line (Fig. 4a) and the SN of rat brains (Fig. 4b,c). Moreover, pKr-2-induced microglial activation and neurotoxicity were significantly diminished in TLR4-deficient mice compared with WT mice (Fig. 5b,c and 6).

We cannot exclude the possibility that another mechanism may be involved in pKr-2-induced neurotoxicity, because TLR4 deficiency did not completely reverse the neurodegeneration induced by pKr-2 treatment (Fig. 6b). In addition, the difference on the microglial activation following to pKr-2 upregulation in human (Fig. 1c) and murine brains (Extended data Fig. 5) is still unclear. However, our observations in the present study show that pKr-2-induced TLR4 overexpression may be an important mechanism for microglial activation, which could contribute to the degeneration of the nigrostriatal DA system in the adult brain. Therefore, our findings strongly suggest that pKr-2 may have a role in the pathogenesis of PD, and the control of pKr-2 expression and pKr-2-induced TLR4 overexpression in microglia may be crucial for protecting the nigrostriatal DA system against PD.

## Materials and Methods

### Institutional review of animal and human protocols

Sprague Dawley (SD) rats (10-week-old) and C57BL/6 mice (8-week-old) were obtained from Daehan Biolink (Eumseong, Korea), and TLR4 KO mice on a C57BL/6 background were kindly provided by Dr. Sung Joong Lee (Seoul National University). All surgical experiments were performed in accordance with approved animal protocols and guidelines established by the Animal Care Committee at Kyungpook National University (No. KNU 2012–37). Human tissue experiments were approved by the Bioethics Committee, Institutional Review Board Kyungpook National University Industry Foundation (IRB Number: 2013–0016), and carried out in accordance with the approved guidelines.

### Materials

Materials were purchased from the following companies: pKr-2 (Haematologic Technologies Inc., Essex Junction, VT; HCP2-0010); rabbit anti-TH (Pel-Freez, Brown Deer, WI; p40101); mouse anti-prothrombin (Haematologic Technologies Inc.; AHP-5013), which recognizes an epitope in the pKr-2 domain of prothrombin; sheep anti-prothrombin fragment 2 (Fitzgerald, Acton, MA; 70R-10587); rabbit/mouse anti-TLR4 (Abcam, Cambridge, UK; ab13556/ab30667, respectively); goat anti-TLR4 (R&D systems, Minneapolis, MN; AF1478); mouse anti-tubulin (Abcam; ab80779); mouse anti-β-actin (Abcam; ab8226); rabbit anti-Iba1 (Wako Pure Chemical Industries, Osaka, Japan; 019–19741); mouse anti-OX-42 (AbD Serotec, Kindlington, UK; MCA275GA); goat anti-IL-1β (Santa Cruz Biotechnology, Santa Cruz, CA; sc-1251); goat anti-TNF-α (R&D systems; AF-410-NA); biotinylated anti-rabbit/sheep IgG (Vector laboratories, Burlingame, CA; BA-1000/6000, respectively); biotinylated anti-mouse IgG (Kirkegaard & Perry Laboratories Inc., Gaithersburg, MD; 16-18-15); Texas Red anti-rabbit/mouse IgG (Vector Laboratories; TI-1000/2000, respectively); FITC anti-mouse IgG (Vector Laboratories; FI-2000); FITC anti-rabbit IgG (Jackson ImmunoResearch Laboratories, Bar Harbor, ME; 711-095-152); horseradish peroxidase (HRP) anti-mouse/rabbit/goat IgG (Invitrogen, Camarillo, CA; 61–6520/65–6120/A24452, respectively); and HRP anti-sheep IgG (GenWay Biotech Inc., San Diego, CA; GWB-3E18C4). The vehicle used to dissolve pKr-2 was sterile PBS.

### Human brain tissues

Frozen and paraffin-fixed brain tissues were obtained from the Victorian Brain Bank Network (VBBN), supported by The Florey Institute of Neuroscience and Mental Health and The Alfred and the Victorian Forensic Institute of Medicine, and funded by Australia’s National Health & Medical Research Council and Parkinson’s Victoria.

### Genotyping of TLR4 KO mice

To confirm the deletion of TLR4, mouse tail biopsies were lysed in DNA digestion buffer [0.1 M Tris-HCl, pH 8.5; 5 mM EDTA, 0.2% sodium dodecyl sulfate (SDS), 200 mM NaCl] with proteinase K added to a 10 mg/mL final concentration and incubated at 55 °C overnight. The lysates were centrifuged at 14,000 rpm and 4 °C for 10 min. The supernatants were transferred to fresh tubes and mixed with isopropanol until the precipitation of DNA was complete. The DNA was dissolved in distilled water and subjected to polymerase chain reaction (PCR) analysis. The PCR primers used for genotyping wild-type TLR4 were 5′-CGT GTA AAC CAG CCA GGT TTT GAA GGC-3′ (forward) and 5′-TGT TGC CCT TCA GTC ACA GAG ACT CTG-3′ (reverse). The temperature cycling conditions were as follows: 2 min at 94 °C; 35 cycles of 94 °C for 30 s, 67 °C for 1 min, and 74 °C for 1 min; and a final extension at 72 °C for 10 min. The PCR product was separated by electrophoresis on a 1.5% agarose gel, stained with ethidium bromide, and then detected under UV light.

### Intranigral pKr-2 injection

Following anesthetization with chloral hydrate, SD rats (230–250 g) or C57BL6 mice (wild genotype and TLR4 KO genotype) were positioned in a stereotaxic apparatus (David Kopf Instruments, Tujunga, CA, USA). Each animal was administered a unilateral injection of pKr-2 into the right SN using the following coordinates: anteroposterior (AP) −0.53 cm, mediolateral (ML) −0.23 cm, dorsoventral (DV) −0.76 cm from bregma for rats; AP −0.32 cm, ML −0.13 cm, DV −0.40 cm from bregma for mice; using a 30-gauge Hamilton syringe attached to an automated microinjector^39^,^40^. The brain volumes between the species are different; therefore, different doses were used for mice and rats. Infusions were delivered at a rate of 0.5 μL/min for pKr-2 (48 μg in 4 μL for rats; 24 μg in 2 μL for mice) or vehicle (sterile PBS) as the control. After infusion, the needle was left in place for an additional 5 min before being slowly retracted. Animals were sacrificed and analyzed at the indicated time points for each experiment.

### pKr-2 treatment of BV-2 microglial cell line cultures

BV-2 microglial cells were plated in 6-well plates and maintained in DMEM supplemented with 5% heat-inactivated FBS and 1% penicillin-streptomycin in 5% CO_2_ at 37 °C. The cells were treated with pKr-2 (50 μg/mL)^7^ or vehicle (4 μl) for 6 h, and then harvested for western blot analysis. This is because the upregulation of inflammatory biomolecules derived from activated microglia was apparent between 3 h and 24 h after pKr-2 treatment^7^.

### Immunohistochemical staining procedures

The postmortem brain sections obtained from the VBBN were deparaffinized and subjected to citrate antigen retrieval prior to immunohistochemistry, and then washed in cold PBS for 15 min, and incubated in blocking solution [0.5% Triton X-100, 1% bovine serum albumin (BSA), 0.05% Tween 20, 0.1% cold fish gelatin, and 0.05% sodium azide in PBS] for 1 h at room temperature. For immunohistochemical staining, the sections were incubated with primary antibodies against pKr-2 (1:200) diluted in 0.1 M PBS containing 0.5% BSA (0.5% BSA blocking buffer) at 4 °C overnight, and then incubated with biotinylated secondary antibodies for 1 h at room temperature, followed by avidin-biotin reagents (Vector Laboratories) for 1 h. The signal was detected by incubating sections in 0.5 mg/mL 3,3′-diaminobenzidine (Sigma, St. Louis, MO) in 0.1 M PB containing 0.003% H_2_O_2_. For immunofluorescence staining, prepared brain sections were rinsed and incubated for 48 h with the following antibody pairs: mouse anti-prothrombin (1:200), which recognizes an epitope in the pKr-2 domain of prothrombin, and rabbit anti-Iba1 (1:1000), or mouse anti-TLR4 (1:200) and rabbit anti-Iba1 (1:1000). The sections were then rinsed and incubated with FITC and Texas Red-conjugated-secondary antibodies (1:200) for 1 h. The stained samples were analyzed under a microscope (Axio Imager; Carl Zeiss, Göttingen, Germany).

Mouse and rat brains were prepared as described previously^39^,^40^,^41^ with some modifications. Animals were transcardially perfused and fixed, and brain sections (30-μm thick) were rinsed in PBS, and then incubated for 48 h with the following primary antibodies: rabbit anti-TH (1:1000), rabbit anti-Iba1 (1:1000), mouse anti-OX-42 (1:500), and rabbit anti-TLR4 (1:200). After incubation, the sections were processed as described above. For Nissl staining, SN tissue samples were mounted on gelatin-coated slides and stained with 0.5% cresyl violet (Sigma).

### Western blot analysis

Animal tissues for western blotting were prepared as previously described^21^,^39^. Briefly, animal SN tissues were removed and sliced using a brain matrix (Roboz Surgical Instrument Co., Gaithersburg, MD). Animal or human tissue samples were homogenized and centrifuged at 4 °C for 20 min at 14,000 × *g*. The supernatant was transferred to a fresh tube and the concentration was determined using a BCA kit. Proteins separated by gel electrophoresis were transferred to polyvinylidene difluoride membranes (Millipore, Billerica, MA) using an electrophoretic transfer system (Bio-Rad Laboratories, Hercules, CA), and the membranes were incubated overnight at 4 °C with the following primary antibodies: rabbit anti-TH (1:1000), rabbit anti-Iba1 (1:1000), mouse anti-OX-42 (1:400), rabbit anti-TLR4 (1:200), goat anti-TLR4 (1:1000), mouse anti-prothrombin (1:100), goat anti-IL-1β (1:200), and goat anti-TNF-α (1:100). After washing, the membranes were incubated with secondary antibodies (1:5000) for 1 h, and then the blots were finally developed with ECL western-blotting detection reagents (Amersham Biosciences, Piscataway, NJ). For quantitative analyses, the density of the bands was measured using a Computer Imaging Device and accompanying software (Fuji Film, Tokyo, Japan), and the levels were quantitatively expressed as the density normalized to the housekeeping protein band for each sample.

### Measurement of dopamine and its metabolites in the STR

Brain tissues for the determination of striatal levels of dopamine and its metabolites were prepared as previously described^20^,^21^ with some modifications. Each brain was placed in a rat/mouse brain matrix, 2.0 mm thick coronal sections were cut through the forebrain, and then the sections were placed flat on a chilled glass plate. The STR sections were dissected using a 2.0 mm tissue punch, and the tissues were frozen immediately on dry ice. Dopamine levels were quantitatively measured using a commercial ELISA kit according to the manufacturer’s instructions (CUSABIO; Wuhan, Hubei, China). Briefly, animal STR issues were homogenized with PBS and stored at −20 °C overnight. After two freeze-thaw cycles, the homogenates were centrifuged at 5,000 × *g* and 4 °C for 5 min. The supernatants were then analyzed using the ELISA kit. The OD of the standards and samples were measured at 450 nm using Softmax software (Molecular Devices, Sunnyvale, CA).

To clarify whether pKr-2 treatment disrupts the nigrostriatal DA system *in vivo*, HPLC was used to measure the levels of dopamine and its metabolites such as DOPAC and HVA in additional rat STR samples, as previously described^20^. Briefly, the isolated tissues were homogenized and centrifuged at 9000 rpm for 20 min in 400 μL of 0.1 M perchloric acid and 0.1 mM EDTA. Then 10 μL samples of supernatant were injected into an autosampler at 4 °C (Waters 717 plus autosampler) and eluted through a μBondapak C18 column (3.9 × 300 mm × 10 μm; ESA Biosciences, Chelmsford, USA) with a mobile phase for catecholamine analysis (Chromosystems, Munich, Germany). The peaks of dopamine and its metabolites were analyzed using an ESA CoulochemII electrochemical detector and integrated using a commercially available software program (Breeze, Waters Corp., Milford, MA). All samples were normalized for protein content as spectrophotometrically determined using the Bio-Rad protein assay kit (Bio-Rad Laboratories).

### Stereological estimation

As previously described^7^,^39^, the total number of TH-ip neurons were counted for the various animal groups using the optical fractionator method performed on an bright field microscope (Olympus Optical, BX51, Tokyo, Japan) using Stereo Investigator software (MBF Bioscience, Williston, VT).

### Quantitative determination of striatal TH immunoperoxidase staining density

Densitometric analysis of the mouse or rat STR was carried out as previously described^21^,^39^ with some modifications. Briefly, an average of 6 coronal sections of the STR were captured at a 1.25× magnification, and the OD was measured using the Science Lab 2001 Image Gauge (Fujifilm, Tokyo, Japan). To control for variations in background illumination, the density of the corpus callosum was subtracted from the density of the STR for each section. TH-ip fiber innervation in the STR was quantitatively expressed as a percentage by comparing the OD on the ipsilateral side with the contralateral control side.

### Statistical analysis

All values are expressed as mean ± standard deviation. Differences between two groups were analyzed using *t*-test. Multiple comparisons among groups were performed using one-way ANOVA and Tukey’s post hoc analysis. All statistical analyses were performed using Sigma Stat software (Systat Software, San Leandro, CA).

## Additional Information

**How to cite this article**: Shin, W.-H. *et al.* Induction of microglial toll-like receptor 4 by prothrombin kringle-2: a potential pathogenic mechanism in Parkinson's disease. *Sci. Rep.* **5**, 14764; doi: 10.1038/srep14764 (2015).

## Supplementary Material

Supplementary Information

## Figures and Tables

**Table 1:** Human postmortem tissues used for immunostaining and western blotting.

Case No.	Diagnosis	PMI (hour)	Age	Tissue
V12/019	Control	25	81.2	SN
03/833		57	79.3	
04/250		31.5	79.6	
07/239		19	78.8	
V12/008	PD	30	81.1	SN
09/292		59	79.9	
10/002		39	79.5	
V12/012		20	79.5	

**PMI**, Postmortem Interval; **SN**, Substantia nigra; **PD**, Parkinson’s disease.
