# Supplementary material for: Induction of microglial toll-like receptor 4 by prothrombin kringle-2: a potential pathogenic mechanism in Parkinson’s disease
Source: Sci Rep. 2015 Oct 6;5:14764. doi: 10.1038/srep14764 (PMC4594003; doi:10.1038/srep14764)
Supplement: Supplementary Information [file srep14764-s1.doc]

Supplementary information

**Induction of microglial toll-like receptor 4 by prothrombin kringle-2: a potential pathogenic mechanism in Parkinson’s disease**

Won-Ho Shin, Min-Tae Jeon, Eunju Leem, So-Yoon Won, Kyoung Hoon Jeong, Sang-Joon Park, Catriona McLean, Sung Joong Lee, Byung Kwan Jin, Un Ju Jung and Sang Ryong Kim

Supplementary information:

Extended Figure Data 1 & Legend

Extended Figure Data 2 & Legend

Extended Figure Data 3 & Legend

Extended Figure Data 4 & Legend

Extended Figure Data 5 & Legend

Extended Figure Data 6 for the cropped western blots shown in the main figures & Legend

**
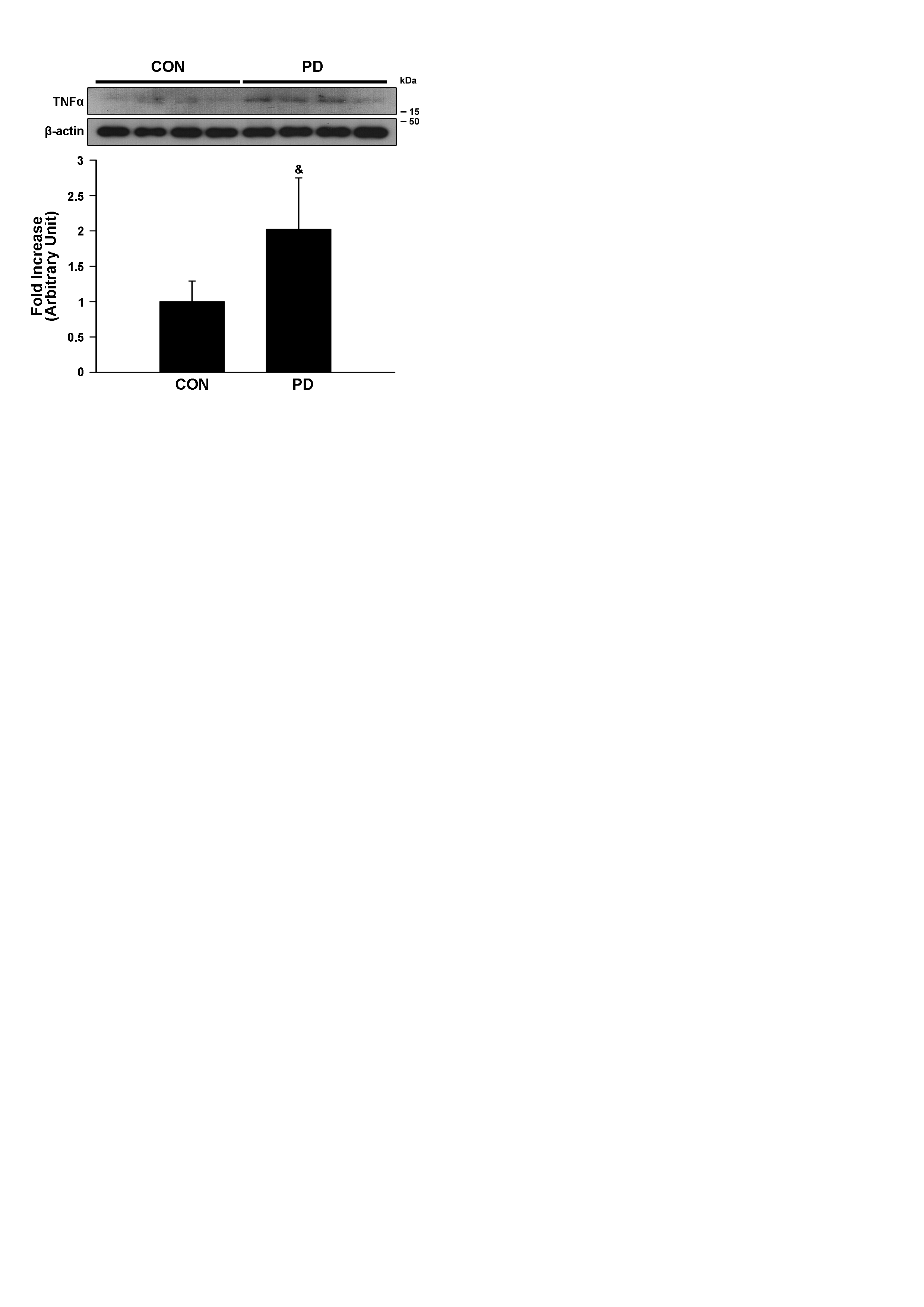
**

**Figure S1. An increase in TNF-α expression in the SN of patients with PD.**

Western blot analysis of TNF-α showed that patients with PD exhibited a significant increase in TNF-α expression in the SN compared with CON. ^&^*p* = 0.040 *vs.* CON (*t*-test; *n* = 4, each group).


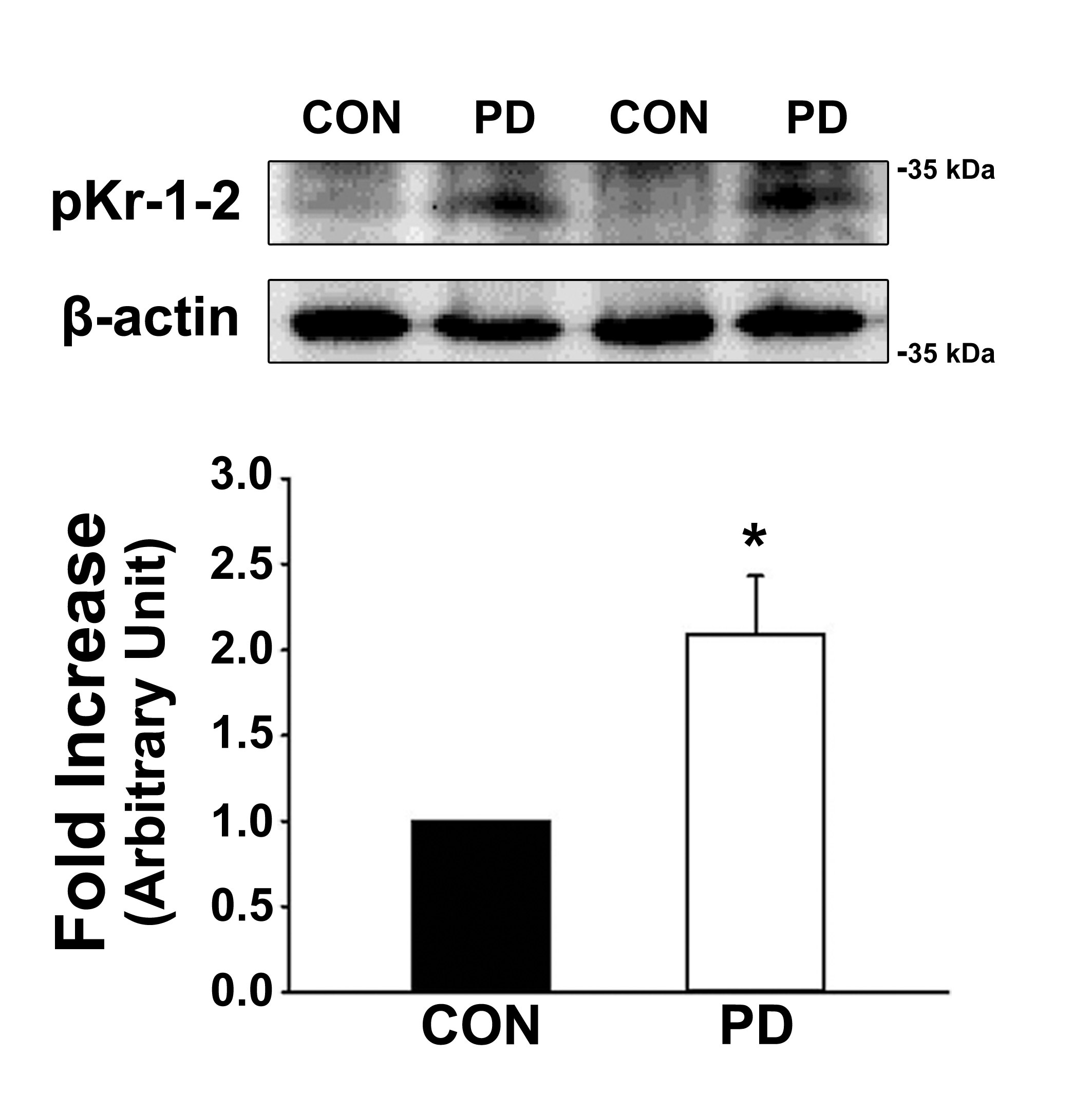


**Figure S2. Induction of pKr-1-2 in the SN of patients with PD.**

Western blot analysis showed that pKr-1-2 expression, which can be cleaved into pKr-1 and pKr-2 by active thrombin, was significantly increased in the SN of patients with PD compared with age-matched controls (CON), suggesting that the an increase in pKr-2 expression might be induced in the SN of PD brains. **p* = 0.018 *vs.* CON (*t*-test; *n* = 4, each group).

**
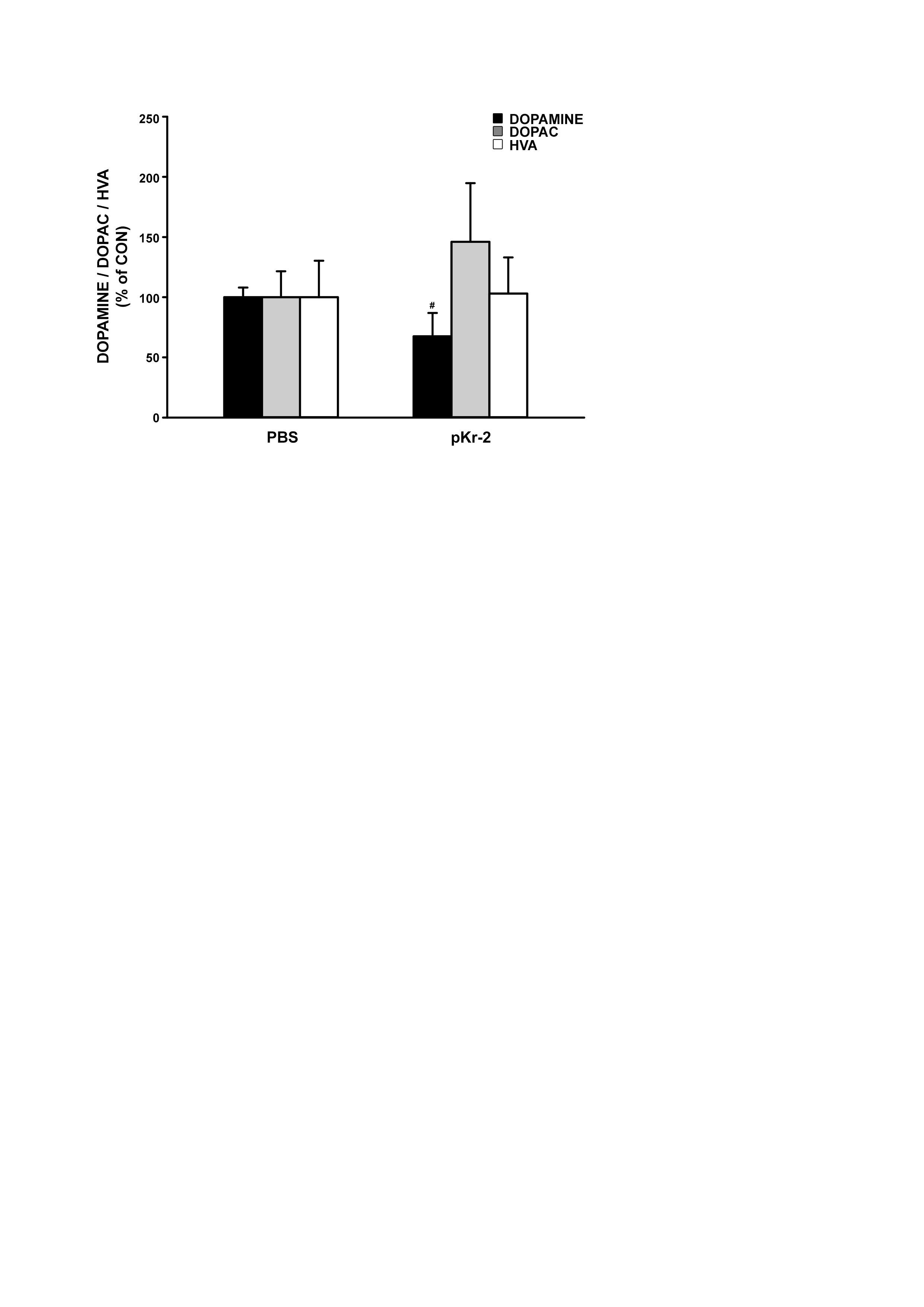
**

**Figure S3. pKr-2 upregulation disrupts the nigrostriatal DA system.**

HPLC analysis of dopamine and its metabolites showed a significant reduction in dopamine levels in the STR of rat brains at 2 weeks after pKr-2 treatment compared with PBS-treated controls (*t*-test; ^#^*p* = 0.044; n = 4, each group). Further, the levels of DOPAC and HVA in the striatum of pKr-2-treated brains were similar to the levels in PBS-treated controls.


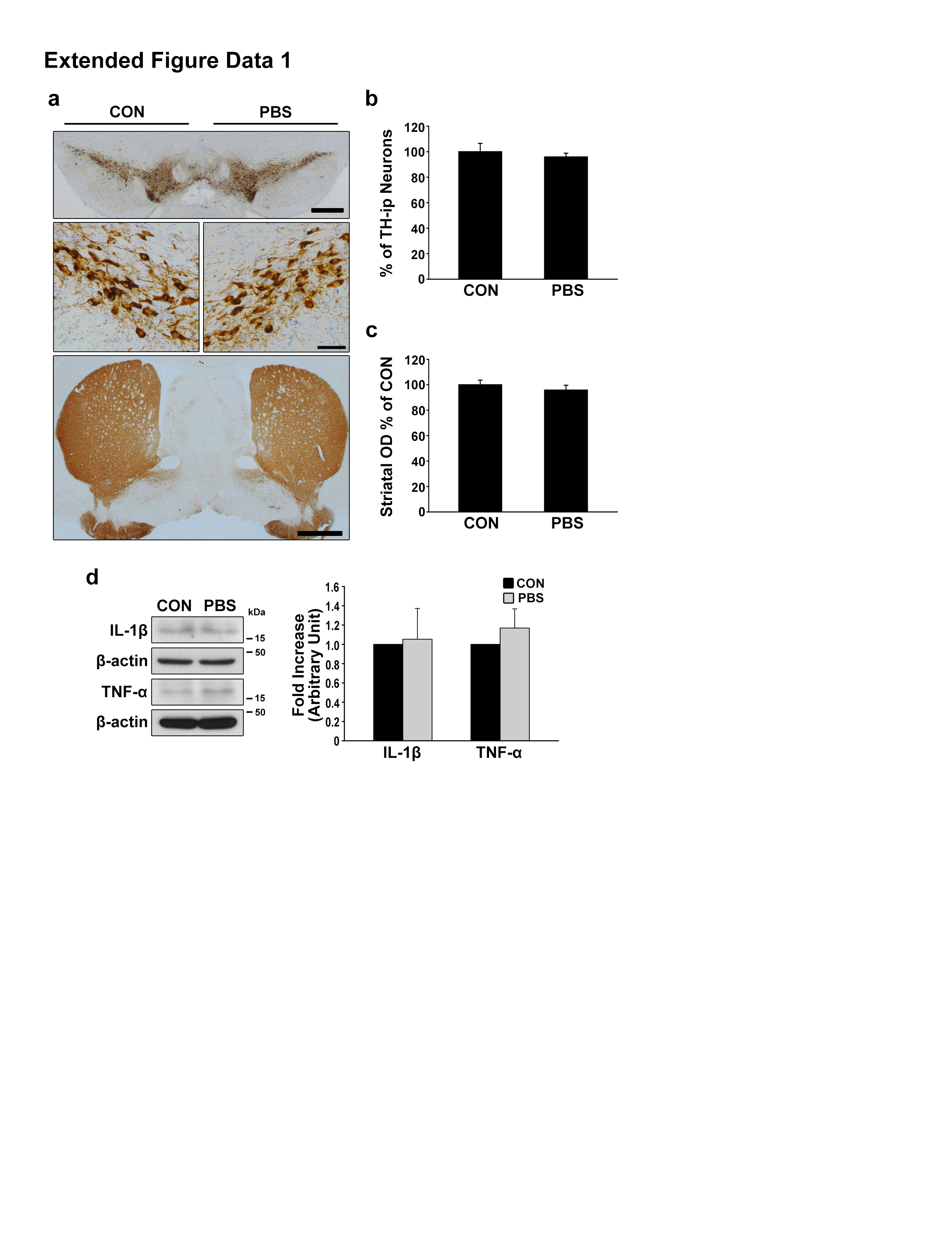


**Figure S4. Effects of PBS on neurotoxicity and microglial activation in the SN of mouse brains.**

(a) C57BL/6 wild-type control mice were administered a unilateral injection of PBS (2 μL) into the SN, and TH immunostaining and Nissl staining were performed one week post-injection. Scale bars, 500 μm and 50 μm, respectively, for the SN, and 1000 μm for the STR. (b, c) Quantitative analysis showed no significant change in the levels of preserved DA neurons and fibers in the SN and STR, respectively, compared with the contralateral untreated control side (*t*-test; *n* = 4, each group). (d) Three days after intranigral injection of PBS, western blot analysis showed no change in the levels of IL-1β and TNF-α in the SN of mouse brains compared with intact controls (*t*-test; *n* = 4, each group).


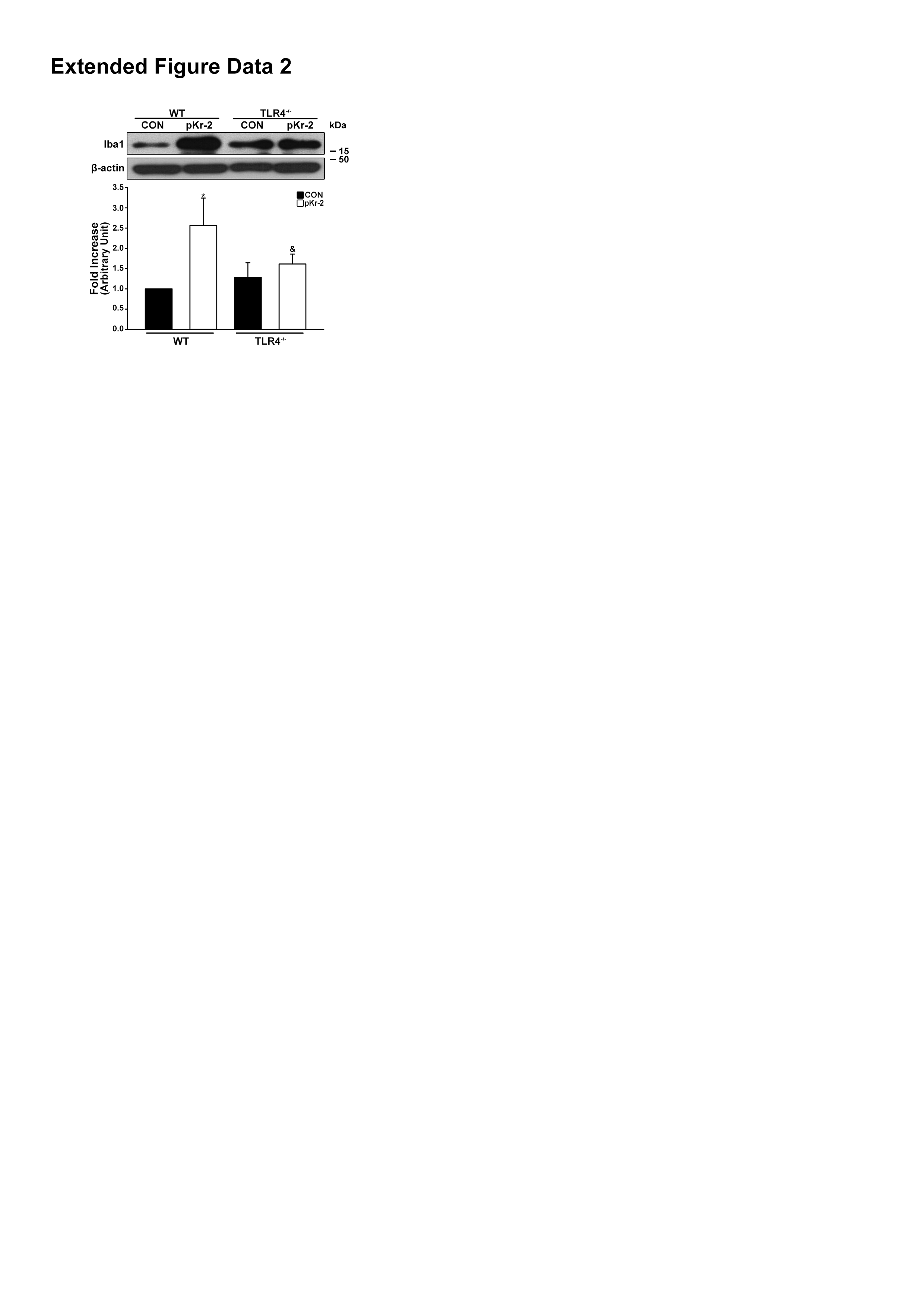


**Figure S5. Changes in Iba1 expression by pKr-2 treatment in the SN of mouse brains.**

At 3 days after injection of pKr-2 (24 μg/2 μl) or PBS as a control, western blot analysis showed a decrease in Iba1 expression in the SN of TLR4 KO mice compared with WT mice. The levels of Iba1 were quantitatively expressed as its OD normalized with that of the β-actin band for each sample. **p* < 0.001 *vs*. PBS-treated mice (CON), and ^&^*p* = 0.009 *vs.* pKr-2-treated WT mice (One-way ANOVA and Tukey’s *post hoc* analysis; n = 4, each group).

**
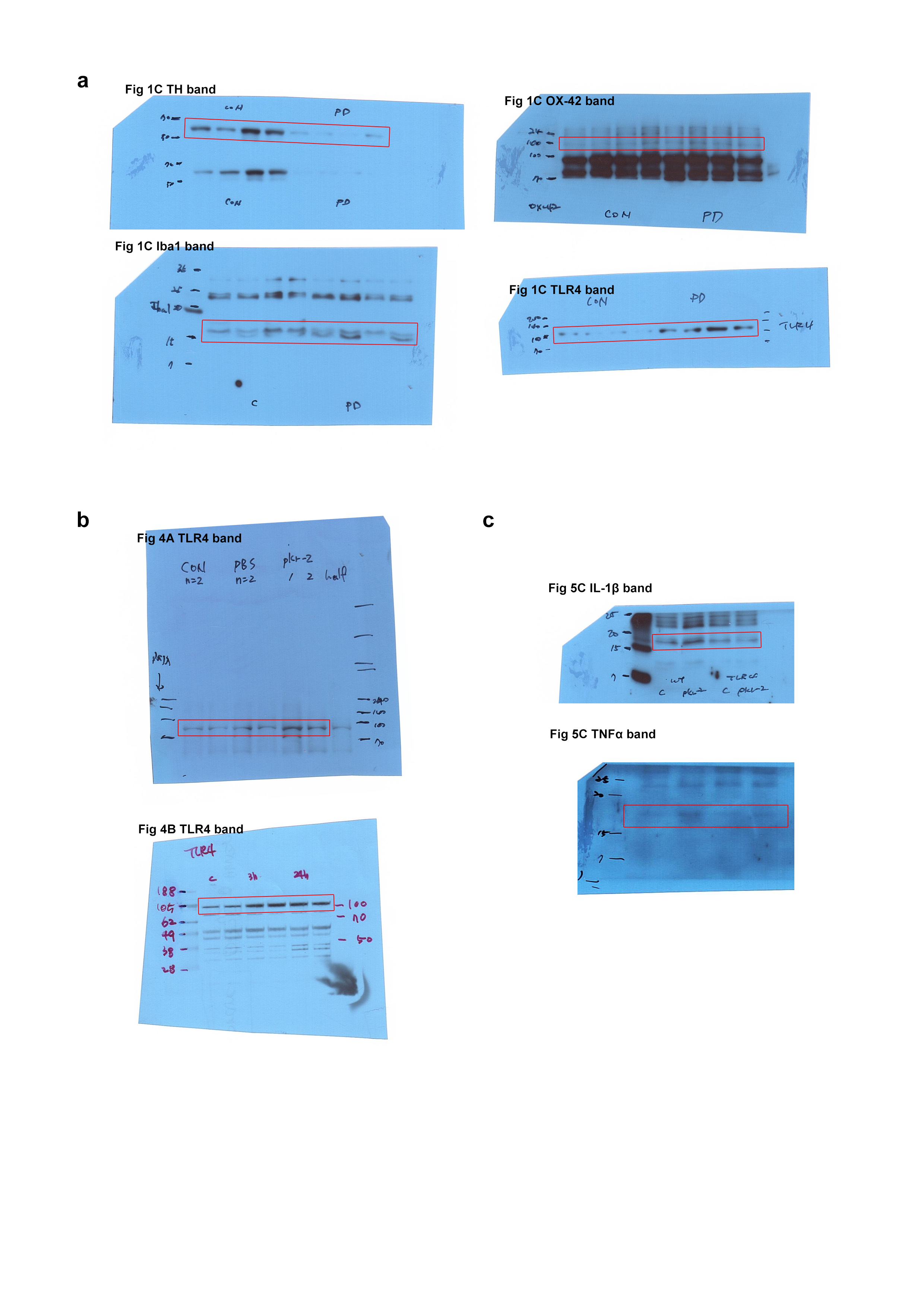
**

**Figure S6. Original blotting results of the cropped western blots shown in the main figures.**

The red rectangles, which are cropping lines in a, b and c, indicate the cropped western blots shown in the Fig. 1, 4 and 5, respectively. To detect the bands under 40 kDa, 15% acrylamide gels for SDS-PAGE were used, and 10% gels for others, and the gels had been run under the same experimental conditions.
